# Effects of Acute Diquat Poisoning on Liver Mitochondrial Apoptosis and Autophagy in Ducks

**DOI:** 10.3389/fvets.2021.727766

**Published:** 2021-08-11

**Authors:** Jiaxin Chen, Yalin Su, Renzhao Lin, Fei Lin, Peng Shang, Riaz Hussain, Dayou Shi

**Affiliations:** ^1^College of Veterinary Medicine, South China Agricultural University, Guangzhou, China; ^2^College of Animal Science, Tibet Agriculture and Animal Husbandry College, Linzhi, China; ^3^Department of Pathology, Faculty of Veterinary and Animal Sciences, The Islamia University of Bahawalpur, Bahawalpur, Pakistan

**Keywords:** diquat, liver, apoptosis, autophagy, ducks

## Abstract

Diquat (DQ) is an effective herbicide and is widely used in agriculture. Due to persistent and frequent applications, it can enter into aquatic ecosystem and induce toxic effects to exposed aquatic animals. The residues of DQ *via* food chain accumulate in different tissues of exposed animals including humans and cause adverse toxic effects. Therefore, it is crucial and important to understand the mechanisms of toxic effects of DQ in exposed animals. We used ducks as test specimens to know the effects of acute DQ poisoning on mechanisms of apoptosis and autophagy in liver tissues. Results on comparison of various indexes of visceral organs including histopathological changes, apoptosis, autophagy-related genes, and protein expression indicated the adverse effects of DQ on the liver. The results of our experimental trial showed that DQ induces non-significant toxic effects on pro-apoptotic factors like BAX, BAK1, TNF-α, caspase series, and p53. The results revealed that anti-apoptotic gene *Parkin* was significantly upregulated, while an upward trend was also observed for Bcl2, suggesting that involvement of the anti-apoptotic factors in ducklings plays an important role in DQ poisoning. Results showed that DQ significantly increased the protein expression level of the autophagy factor Beclin 1 in the liver. Results on key autophagy factors like LC3A, LC3B, and p62 showed an upward trend at gene level, while the protein expression level of both LC3B and p62 reduced that might be associated with process of translation affected by the pro-apoptotic components such as apoptotic protease that inhibits the occurrence of autophagy while initiating cell apoptosis. The above results indicate that DQ can induce cell autophagy and apoptosis and the exposed organism may resist the toxic effects of DQ by increasing anti-apoptotic factors.

## Introduction

**Figure 1:**
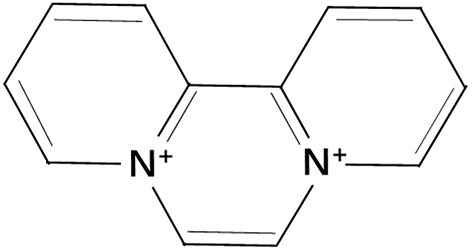
The molecular structure of DQ.

Diquat (1,10-ethylene-2,20-bipyridinium, DQ) is a widely used non-selective herbicide that belongs to bipyridine (Figure 1) (1, 2). The toxicity of DQ is less different than that of other herbicides like paraquat (PQ) due to its rapid degradation in the environment. Although DQ is less toxic than other herbicides, there are still numerous adverse effects that are related to DQ. A previous report has indicated that accidental exposure to this herbicide during applications induces different toxic effects (3). In addition, a previous study has shown that long-term exposure to DQ can increase the risk of Parkinson's disease in animals including public health (4). DQ can easily enter into aquatic ecosystem *via* direct discharge from production industry, agricultural sector, and runoff (5) and causes serious threats to fish (6), freshwater snails (7), ducks (8), and other aquatic animals. Peking ducks are mainly raised in rural areas of China, which are meat-based species. We choose the Peking ducks as our experiment animals because these animals have the closest connection with humans.

The liver is the target organ of DQ toxicity. The mechanism of liver injury induced by DQ is complex, but it is mainly related to reactive oxygen species (ROS). DQ can induce increased oxidative stress to produce a large number of superoxides and can alter different liver functional activities such as metabolism, detoxification, and immune response resulting in the production of a large number of ROS in the liver (9). The liver toxicity leads to hepatocyte degeneration, inflammation, mitochondrial dysfunction, and even apoptosis (10, 11). Oxidative stress can reduce glutathione (GSH) in the liver and can enhance the production of ROS to promote the activation of pro-apoptotic factors such as p53 and Bax leading to apoptosis (12). In the liver, the mitochondria are also affected by overproduction of ROS ultimately leading to oxidative stress. Studies have indicated that DQ can decrease the activities of mitochondrial complex I, II, III, and V in the liver and decrease the abundance of mitochondrial biosynthesis its related genes (*SIRT1, PGC1ATP*, and *TfAM*). Furthermore, oxidative stress can affect the activities of different antioxidant enzymes and interfere with the antioxidant defense mechanisms (13) in the liver and blood. It is reported that infants or piglets with underdeveloped antioxidant systems are more vulnerable to oxidative damage and liver diseases (14). The mechanisms of toxicity of DQ are still under debate. However, DQ induces its toxic effects in the liver through a variety of liver injuries.

Apoptosis is a process of cell death regulated by different genes, while autophagy is a self-protective process of degradation of its own components through the lysosomal system. Herbicides such as DQ and PQ can induce abnormal mechanisms of apoptosis and autophagy. A previous study has shown that different toxins can induce oxidative stress in mice and produce a large amount of ROS to promote DNA damage, upregulation of genes regulating cell cycle (CDKN1A, CDKN2C, and CDKN2D), and pro-apoptotic genes (*caspase-3* and *Bax*) eventually leading to promote apoptosis (15). Nitrite interferes with the expression of anti-apoptosis gene *Bcl2* and leads to apoptosis (16) by enhancing the expression of *caspase-3* and *Bax* genes in tissues. It has been recorded that aloe emodin can activate abnormal signals of apoptosis in the liver of zebrafish, promote the expression of p65, upregulate the proteases caspase-3 and Bax in p53 pathways while can downregulate Bcl2 in p53 pathway, and upregulate the expression of TNF-α and JNK in NF-κB that can induce inflammation and apoptosis (17). Different insecticides like fipronil can also promote the production of peroxisome that damages the DNA and mitochondria, causes the overexpression of Bax, and aggravates hepatocyte apoptosis (18). Furthermore, studies have reported that various drugs can cause hepatocyte apoptosis. The most commonly used anti-tuberculosis drug activates inflammatory corpuscles NLRP3 to upregulate the expression of p53, Bax, and Cleaved-Cas3, inhibit the expression of Bcl2, induce inflammation and apoptosis, and damage hepatocytes in rats (19). High concentrations of acetaminophen can reduce GSH resulting in increased oxidative stress and severe mitochondrial dysfunction, promoting the expression of key apoptotic factors, and promoting apoptosis (20, 21). A previous study has shown that increased concentrations of manganese (Mn) can induce neurotoxicity, increase the levels of glutathione peroxidase (GSH-Px), superoxide dismutase (SOD), and malondialdehyde (MDA), and promote the expression of p53, Bax, Bak, fas, and caspase-3 to induce apoptosis (22). A previous study has reported that cadmium affects the expression of IP3R1 receptor through Ca^2+^ channels, induces DNA damage, and promotes the abnormal process of autophagy or apoptosis (23). Arsenic (As) and its methylation metabolites not only affect regulatory enzymes such as 2 (ERK2), p38, or c-jun to induce neuronal and neuroblastoma cell apoptosis through MAPK signaling pathways but also induce apoptosis through AMP-dependent protein kinase (AMPK)/mTOR signal pathways (24). The morphology of the mitochondria is also important in apoptosis (25, 26). The related proteins Drp1, Mfn1, and Mfn2, mitochondrial a-KGDH, that affect cell division and fusion are all related to the mitochondria (27). The mitochondria play a central role in the integration and circulation of intracellular death signals, such as oxidative stress and DNA damage (28, 29).

The mitochondria can cause cell injury or apoptosis by producing ROS, pro-inflammatory signals, or through mitochondrial membrane permeability. A little electron may escape from the mitochondrial electron transport chain resulting in the production of superoxide. The increased oxidant load also promotes the extra ROS produced by mitochondrial complex I to further enhance cell oxidative stress and promote cell death (30). In addition, ROS produced by the mitochondria can activate NLRP3, adaptation proteins ASC, and caspase-1 to form inflammatory bodies, and the accumulation of damaged mitochondria aggravates inflammation and leads to cell damage. The change of mitochondrial permeability is also an important cause of cell death, which leads to the dissipation of mitochondrial transmembrane potential and the cessation of oxonase. Furthermore, it leads to rapid necrosis of apoptotic (31). When apoptosis is induced by mitochondrial damage, the decrease of ATP produced by the mitochondria through respiratory chain or the increase of ROS will lead to the increase of autophagy (32). Moreover, the induction of autophagy affects the circuit in which the mitochondria transmit lethal signals, protecting cells from other deadly stimuli (33). However, the relationship between autophagy and apoptosis affected by the mitochondria is complex. These two pathways are activated at the same time and can be regulated by the same factors such as Bcl-2 family proteins, cystatin (caspase), ATG proteins, and p53. On the other hand, autophagy and apoptosis are antagonistic to each other, and autophagy of damaged mitochondrial cells can reduce apoptotic signal transmission and protect normal cells.

The pathways through which DQ induces toxic effects are not clear. Therefore, it is interesting to speculate that DQ may induce harmful effects *via* induction of oxidative stress and increased level of apoptosis factors by regulating apoptosis and autophagy-related signal pathways like NF-κB, MAPK, and mTOR (34). Autophagy is beneficial to inhibit DQ-induced apoptosis and alleviate the effects of DQ poisoning (35). Therefore, the purpose of this study was to explore the relationship between liver injury and apoptosis and autophagy genes and proteins in ducks induced by acute DQ poisoning.

## Materials and Methods

### Animals and Treatment

All the experiments were approved by the Animal Ethics Committee of South China Agricultural University (License Number: 2017A087) and were conducted following the ethical code of conduct for animal care and use. After 7 days of acclimatization, a total of 60 1-day-old Peking ducks were randomly divided into two groups including the control and treatment groups (feeding 100 mg/kg DQ on the first day). The ducks in the treatment group were exposed to DQ for 11 days. After weighing at day 11, ducks were anesthetized by injecting chloral hydrate through the intraperitoneal route. Blood was collected from the jugular vein for biochemical profile. The heart, liver, kidney, thymus, spleen, and bursa of fabric of the ducks were removed, weighed separately, and photographed, and the coefficient of each organ was calculated.

### Histopathological Examination

Liver tissue of 1 × 1 cm was cut and fixed in 4% paraformaldehyde solution. After that, all the tissues were processed and then embedded in paraffin for 24 h. Approximately 0.4 μm thick liver slices were obtained with the help of KEDEE KD2258 manual rotary microtome and were then stained with hematoxylin and eosin (H&E). Briefly, the paraffin slices were dewaxed in xylene solution for 20 min and then sequentially soaked in benzene alcohol, anhydrous ethanol I, anhydrous ethanol II, 95% alcohol I, 95% alcohol II, and 80% alcohol solution each for 10 min to remove xylene. After that, the slices were soaked in hematoxylin for 8 min, rinsed with water for 15 min, then shaken in the differentiation solution for ~2 s, rinsed with water for 20 min again, and dyed again with eosin for 8 min. The dehydration and transparency procedures used in paraffin sections preparation were repeated. The residual liquid on the slice was removed, and neutral resin was dropped on slice, then sealed, and baked for more than 6 h. Finally, all the prepared sections were observed under light microscope (Y-TV55; NiKon, Japan) and photographed.

### RT-qPCR Analysis

The primer and sequences used for real-time PCR (RT-PCR) are shown in Table 1. Total RNA was lysed from 100 mg of the liver using RNA isolate reagents (Vazyme, China). Total RNA was separated with chloroform and precipitated with isopropanol. Ethanol was used to wash the residual isopropanol. Total RNA concentration was measured by Microvolume UV–Vis spectrophotometer (Nanodrop™ One; Thermo Fisher Scientific, Madison, WI, USA). Approximately 5 μg total RNA was reverse-transcribed into cDNA using HiScript III RT SuperMix for qPCR (Vazyme, China). The mixture contained 1 μl cDNA primer, and ChamQ University SYBR qPCR Master Mix (Vazyme, China) was used to perform RT-qPCR on the real-time PCR detection system (QuantStudio™ 5; Thermo Fisher Scientific, Waltham, MA, USA). The 2^−ΔΔ(*Ct*)^ method was used to calculate the relative gene expression level, and GAPDH was used as the internal reference gene. The results are expressed as normalize mRNA levels by reference gene.

**Table 1:** Primer sequences used for real-time PCR.

**Gene**	**5^**′**^-primer (*F*)**	**bp**	**3^**′**^-primer (*R*)**	**bp**
*COL2A1*	GAGCGGAGACTACTGGATCG	20	TTCTTGTCTTTGGCCTTGCT	20
*BAK1*	CCGCTACCAACAGGAGAGAG	20	GCGTCGTACCGCTTGTTAAT	20
*BAX*	CTTCTGCTTCCAGACCAAGG	20	TCAGCGTGTTCTTCCTGTTG	20
*Bcl2*	GAGTTCTCCCGTCGCTACC	19	CGGTTCAGGTACTCGGTCAT	20
*Caspase-3*	CGGGTACGGATGTAGATGCT	20	GGGGCCATCTGTACCATAGA	20
*Caspase-9*	GAACTGGATCCGATGTGGAC	20	TTCCGTCCGTTCCATAAATC	20
*P53*	ACAGCAGACTCCTGGGAAGA	20	GGGGTATTCGCTCAGTTTCA	20
*LC3A*	GCTGGACAAGACCAAGTTCC	20	ACCCTCCCTGGACAGAAAGT	20
*LC3B*	TTCGAGAGCAGCATCCTACC	20	CCTTCTCGCTCTCGTACACC	20
*P62*	GGACCCACTTGTCTTCCAAA	20	AGCCTCTCGCAGTCCTGTAG	20
*Parkin*	TGATGGGCTTTGTGAAATGA	20	TTCAGCGTGACACAGAGGAC	20
*GAPDH*	GGTAGTGAAGGCTGCTGCTGATG	23	GGAGGAATGGCTGTCACCGTTG	22

### Western Blot Analyses

The antibodies used for Western blot are shown in Table 2. Approximately 80 mg of the liver was lysed in RIPA lysis buffer (Meilunbio, China) and 1 mM protease inhibitor (PMSF) (Meilunbio, China) at 4°C, and the concentration was determined using the BCA protein concentration determination kit (Beyotime, China). After that, samples were diluted with 5 × SDS-PAGE loading buffer and boiled for 8 min. An equal amount of protein sample (10 μg) was added and electrophoresed on a 12.5% SDS-polyacrylamide denaturing gel and then transferred to polyvinylidene fluoride membranes. After blocking with Tris buffered saline Tween (TBST) containing 5% skimmed milk powder for 1 h, primary antibodies were incubated with membranes for 16 h. After washing with TBST for three times, the membranes were blocked with secondary antibody for 1 h. Finally, an electrochemiluminescent liquid (ECL) (Meilunbio, China) was prepared to measure the signal imprinting. ImageJ software was used to calculate the gray value of each band and perform normalization processing.

**Table 2:** Antibodies used for Western blot.

**Name**	**Company**	**Cat. no**.	**Concentration**
Beclin 1 Rabbit pAb	ABclonal	A17028	1:1,000
LC3B Rabbit pAb	ABclonal	A11282	1:1,000
SQSTM1/p62 Rabbit pAb	ABclonal	A11483	1:1,000
GAPDH Rabbit pAb	ABclonal	AC001	1:9,000
Goat anti-Rabbit IgG (H&L)	Zenbio	511203	1:5,000

### Statistical Analysis

Statistical analysis was performed on all data using GraphPad Prism 8.0 (GraphPad Inc., La Jolla, CA, USA) and SPSS for Windows (version 22; SPSS Inc., Chicago, IL, USA). The independent sample *t*-test was used to analyze the differences of the data between each group. Data were expressed as the mean ± standard deviation (SD). The data between different groups were analyzed by one-way analysis of variance (ANOVA) (*n* = 2, each repeated three times). Significance level was considered as *p* < 0.05, *p* < 0.01, and *p* < 0.001.

## Results

### The Influence of DQ on Organ Index and Serum Biochemistry

**Figure 2:**
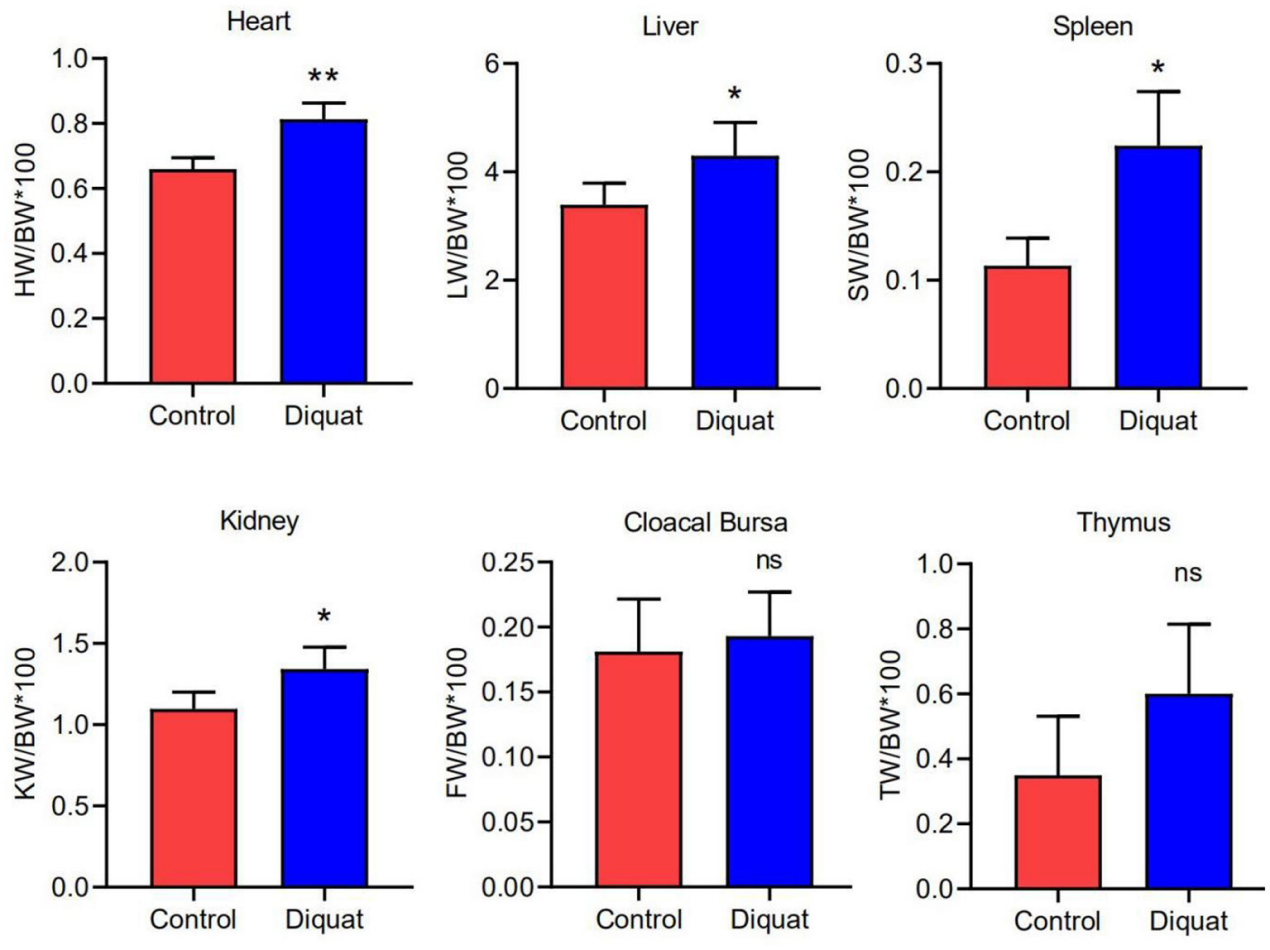
The organ index of the heart, liver, spleen, kidney, cloacal bursa, and thymus. Error bars indicate standard error of the mean (*n* = 4). “ns” and “*” indicate the level of significance. (“ns” means no significant difference, *p < 0.05, **p < 0.01 compared with the control conditions).

The indexes of the heart, liver, kidney, and spleen were significantly higher in treated ducks (Figure 2) than in the control group (*p* <0.05). The results showed no significant difference in the index of the cloacal bursa and thymus (*p* > 0.05) as compared with ducks of the control group.

The results of serum biochemical examination are shown in Table 3. The results showed that DQ reduces the serum levels of calcium (Ca), phosphorus (P), triglyceride (TG), total protein (TP), total cholesterol (TC), albumin (ALB), γ-glutamyl transpeptidase (γ-GT), alkaline phosphatase (ALP), aspartate aminotransferase (AST), and total bilirubin (T-Bil) as compared with the control group. Among them, Ca, TG, ALB, ALP, and T-Bil are significantly reduced (*p* < 0.05), and AST is extremely significantly reduced (*p* < 0.01).

**Table 3:** Serum biochemical index.

**Parameter**	**Units**	**Control (*n* = 3)**	**Diquat (*n* = 3)**	***p***
Ca	Mmol	1.88 ± 0.20	1.35 ± 0.09	<0.05
P	mmol	2.49 ± 0.92	1.70 ± 0.21	ns
TG	mmol	1.00 ± 0.32	0.37 ± 0.13	<0.05
TC	mmol	7.81 ± 2.79	4.74 ± 0.44	ns
TP	g/L	22.03 ± 3.84	18.27 ± 0.87	ns
T-Bil	μmol/L	50.57 ± 11.27	27.57 ± 6.14	<0.05
γ-GT	U/L	4.47 ± 1.05	2.97 ± 0.67	ns
ALP	U/L	1,459 ± 223.70	891.90 ± 191.20	<0.05
ALT	U/L	64.27 ± 2.30	77.30 ± 16.52	ns
AST	U/L	54.20 ± 1.02	35.07 ± 2.83	<0.001
ALB	g/L	7.97 ± 0.90	6.20 ± 0.36	<0.05
GLU	mmol	7.21 ± 2.43	8.18 ± 1.50	ns
γ-GT/ALT		0.070 ± 0.018	0.038 ± 0.001	<0.05

“*ns” indicates the level of significance, and it means no significant difference*.

### The Effect of DQ on Liver Histopathology

**Figure 3:**
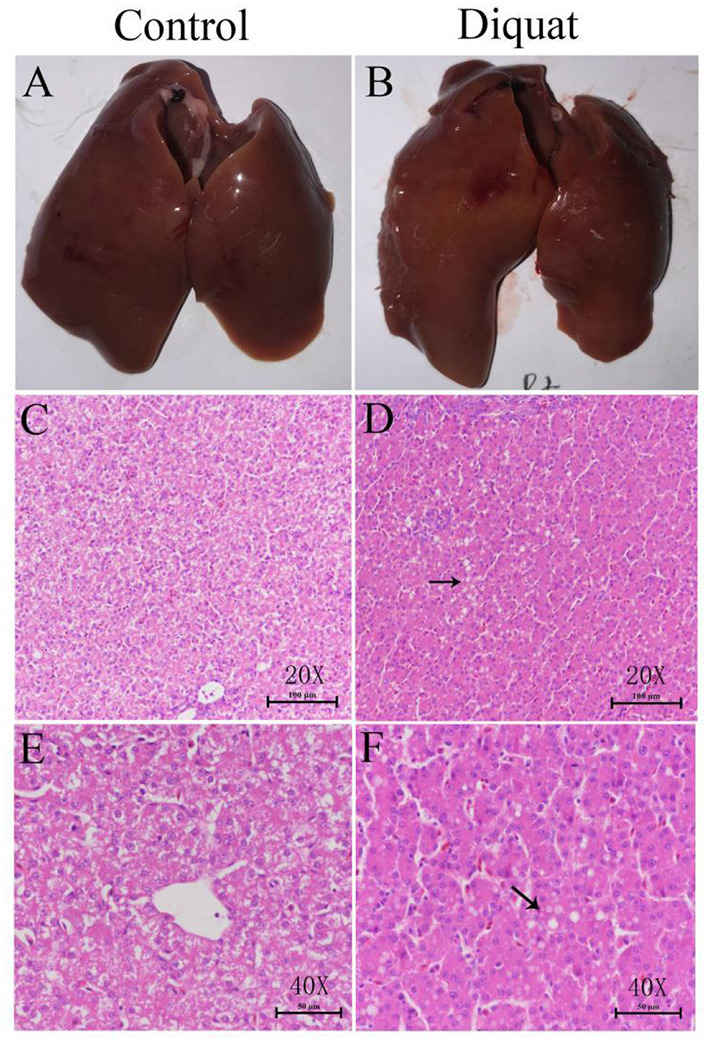
The effect of diquat on the liver. **(A)** The liver of the control group. **(B)** The liver of the DQ treated group. **(C)** Control group (HE, 20**×**). **(D)** DQ treated group (HE, 40 ×). **(E)** Control group (HE, 40 ×). **(F)** DQ treated group (HE, 40 ×).

In general, there were no obvious pathological changes in the liver of treated and untreated control groups (Figure 3). The basic liver structure of the control group and the DQ treatment group was normal and clear. No inflammatory response was observed in the portal area and liver parenchyma. Histologically, the liver cells of the control group were slightly enlarged, and the accumulation of eosinophilic glycogen material was observed in the cytoplasm. In the DQ treatment group, a large number of vacuoles (shown by black arrows) were observed in the liver cells, which were diffusely distributed. In liver sections of DQ, treated duck's fatty infiltration was obvious indicating that the drug may cause liver fat metabolism disorders.

### The Effect of DQ on Cell Apoptosis

**Figure 4:**
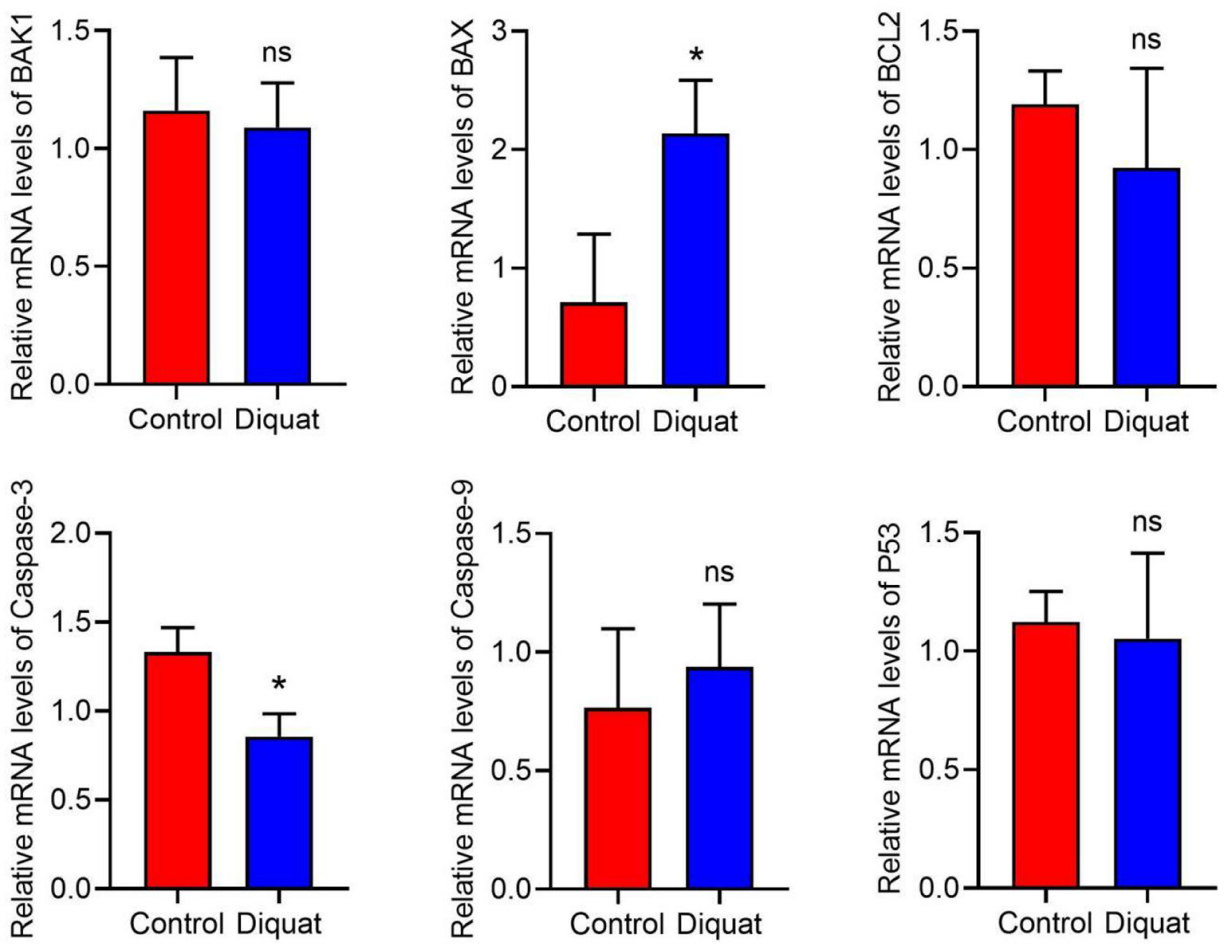
The expression of apoptotic genes in the liver. DQ affects the mRNA expression levels of apoptosis-related genes in liver, including BAK1, BAX, Bcl2, Caspase-3, Caspase-9, and P53. “ns” and “*” indicates the level of the Significance. (“ns” means no significant difference, *p < 0.05 compared to the control conditions).

Compared with the control group, the expression of apoptosis-related genes is recorded as indicated in Figure 4. The expression level of caspase-3 decreased significantly, and the expression of Bax increased significantly. In addition, there was no significant difference in the expression of *Bak1, Bcl2, p53*, and *Caspase9* genes. In these genes, only *Caspase9* showed an upward trend, and the others showed a downward trend.

### The Effect of DQ on Autophagy

**Figure 5:**
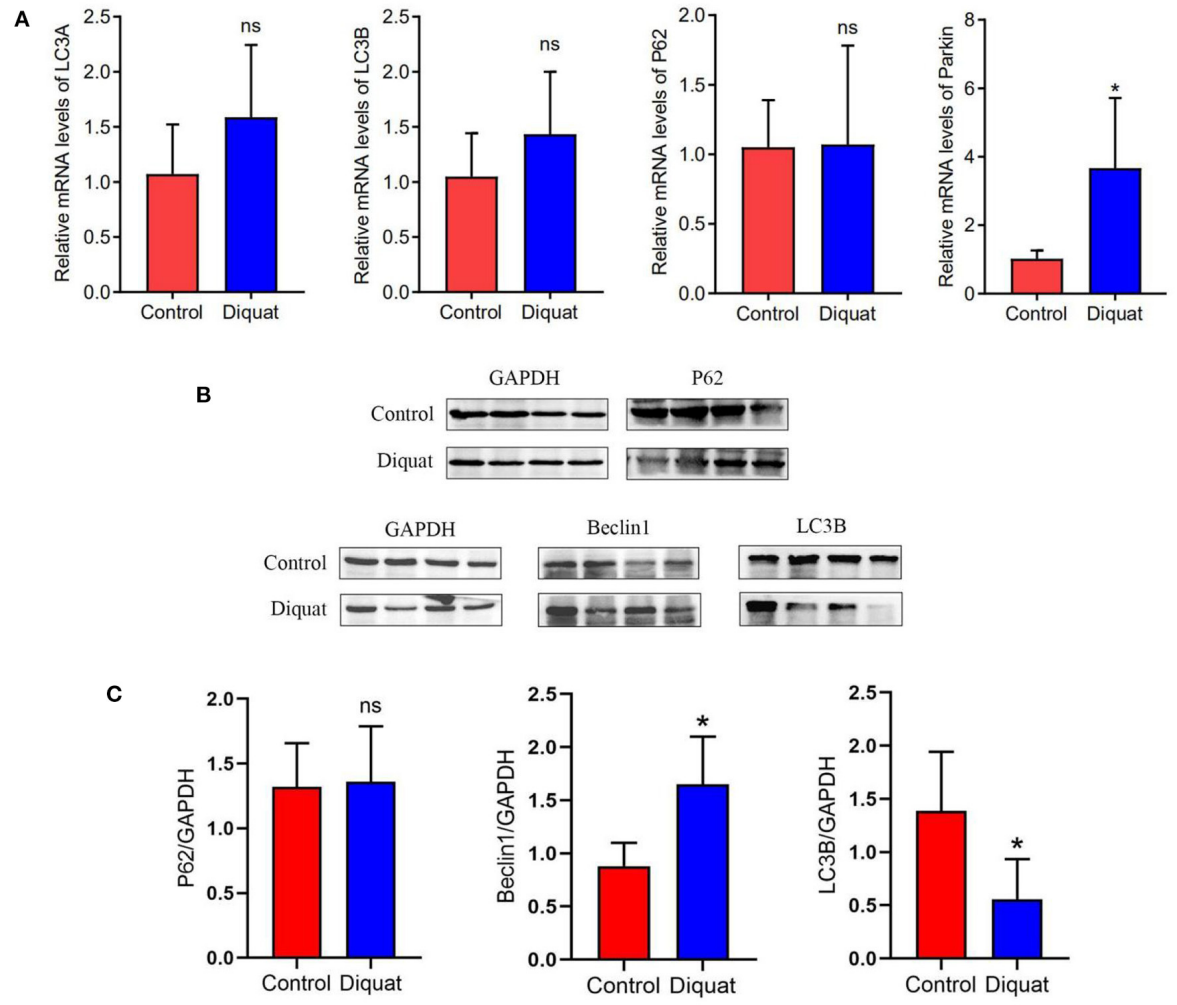
The expression of autophagy genes and proteins in the liver. **(A)** Autophagy mRNA gene expression: LC3A, LC3B, and P62. **(B)** Western blot detects protein expression bands: P62, Beclin 1, and LC3B. **(C)** The level of the protein expression: P62, Beclin 1, and LC3B. “ns” and * indicates the level of the significance. (“ns” means no significant difference, *p < 0.05 compared to the control conditions).

The changes of autophagy-related genes and proteins in the ducks treated with DQ are shown in Figure 5. As compared with the control group, there was no significant difference in the expression of *LC3A, LC3B*, and *p62* genes, in which *LC3A* and *LC3B* increased non-significantly, and E3 ubiquitin ligase gene *Parkin* increased significantly. The protein expression like LC3B decreased significantly, while Beclin 1 increased significantly.

## Discussion

Different histopathological changes in liver sections of treated ducks were observed in our experimental study. Previously, various pathological lesions such as widening of the alveolar septum with inflammatory cells, narrowing and atrophy of the alveolar sac and proliferation of collagen fiber in the lungs, and swelling and necrosis of renal tubular epithelial cells and telangiectasia in the kidneys have been reported (36). Inflammation of cardiomyocytes accompanied by inflammatory cell infiltration in the heart and focal inflammatory cell infiltration in the brain tissue have also been reported (37). In DQ exposed patients, the values of serum creatine and urea nitrogen were significantly increased suggesting severe renal function abnormalities (38). Mild bilateral patchy consolidation at the hilar in the lungs has been observed (39). The lungs mainly involve the alveolar epithelium, which may initially cause acute alveolitis and then pulmonary fibrosis (34). The inflammatory spots around the pleura gradually expand into plaques over time and developed into pulmonary fibrosis (40). Studies have found that the injection of DQ in pigs can increase the expression of mitochondria-related proteins including Pink1, Parkin, and LC3B in the intestine. It is suggested that DQ can induce mitochondrial autophagy (41). Previously, it has also been found that DQ can reduce the activity of SOD and GSH-Px in the intestinal mucosa and increase the contents of MDA (42, 43), suggesting that PQ causes oxidative stress in the intestine. *In vivo* and *in vitro* studies have shown that the oxidative stress caused by DQ can damage the integrity of the intestinal epithelial barrier that is manifested by the destruction of tight junctions (TJ) and reduction of epithelial cell viability (44–47). Different studies have indicated manifestations of DQ toxicity as decreased TER and increased FD4 flux (48), high serum lipopolysaccharide levels, and diamine oxidase activity in piglets (43). DQ reduces the activity of AHR in the spleen, causing oxidative damage to the spleen. The decreased activity of SOD and CAT suggests decreased antioxidant functions in the spleen (49). Long-term exposure to DQ had toxic effects on the reproductive system (50–52) that is manifested by reducing the quality of germ cells affecting early embryo development (53). Long-term ingestion of DQ in rats and dogs can induce cataracts (54). Dose-dependent axonal degeneration has been observed in dorsal root ganglion neurons (55). In addition, the production of nitric oxide and superoxide anion free radicals was significantly increased, and lipid peroxidation increased, suggesting that PQ causes neurotoxicity (56).

The central vein around the liver cells showed vacuolar degeneration with punctate necrosis due to exposure to DQ (36). The activity of total antioxidant capacity, SOD, and GSH-Px in the liver of piglets injected with DQ decreased suggesting lower status of the liver's antioxidant capacity. There were significant differences in the expression of liver mRNA and lncRNA. GNMT is highly expressed, and GCK is downregulated. These results indicate that DQ can affect glucose metabolism in the liver and reduce weight gain (57). It can also increase the accumulation of glutathione peroxidase (GPX) activity and MDA in the plasma and liver; increase the activity of AST, alanine aminotransferase, and T-Bil concentrations; and increase the relative liver weight, indicating that DQ causes liver damage (10). DQ increases Ca efflux due to the weakened ATP-dependent Ca chelation of liver microsomes (58). DQ-induced liver lipid peroxidation is manifested as increase in 11-, 12-, and 15-hydroxyeicosatetraenoic acid. DQ can also activate inflammatory cells, leading to the synthesis and release of certain pro-inflammatory cytokines like TNF-α, IL-1β, and IL-6 (59).

As a commonly used herbicide, DQ mainly destroys plant cells to achieve the purpose of weeding by inducing redox cycle, releasing ROS and nitric oxide and inhibiting the effect of NADPH. However, it is the herbicidal principle that causes serious damage to the nervous system, liver, kidney, heart, and lungs of animals or humans, while the liver is the main source of ROS, and it has become the target of DQ (60). Liver injury has a prodigious impact on the organism, which can directly lead to an increase in morbidity and mortality of terrestrial and aquatic animals, especially birds (61).

Many studies have been conducted on DQ-induced liver injury, which mainly focus on oxidative stress, apoptosis, and autophagy. Previous studies have shown that DQ induces liver redox cycle to produce superoxide while inhibit the production of antioxidant enzymes, so that antioxidant enzymes are not enough to resist liver damage caused by oxidation. Secondly, the endoplasmic reticulum and mitochondria can regulate apoptosis and autophagy, while damaged mitochondria initiate autophagy mechanism to inhibit the release of cytochrome c (Cyt-c) and induce apoptosis. Damaged mitochondria can also reduce the accumulation of ROS, which inhibit the division of the mitochondria and prevent it from degradation by autophagy (62).

In the specific effect of DQ on the liver, some scholars have found that the content of glutathione disulfide (GSSG) can reflect the liver injury induced by DQ, while previous studies have shown that DQ leads to a sharp increase in the content of GSSG in the liver (12, 63). DQ can also increase the level of serum ALT and AST and decrease the level of anti-apoptosis factor Bcl2, which indicates hepatocyte apoptosis and injury (64). When DQ is put into the waters of fish fry, it was found that biosynthesis of protein and RNA was increased, while ATK/mTOR signal, SREBP pathway, and caspase pathway were activated. It was considered that this change was closely related to the increase of protein and mRNA, which further confirmed the occurrence of oxidative stress and apoptosis in fish (65–67). It is also found that intraperitoneal injection of DQ in piglets affects the MAPK signal pathway that leads to acute oxidative stress and increase lipids, antioxidant metabolites, and peroxide MDA in the liver (68), so it indicates that DQ can destroy cell lipids and induce cell death. Some scholars have found that DQ-induced oxidative stress can promote the production of H_2_O_2_ in the mitochondria, depolarize the mitochondria, and inactivate iron and sulfur in the mitochondria containing aconitase and other proteins, resulting in mitochondrial damage (69–72). Previous studies have found that mitochondrial damage and induced mitochondrial release of apoptosis and oxidation-related proteins can promote apoptosis. Oxidative stress induces the release of Cyt-c from the mitochondria that is the key factor of apoptosis, while the antioxidant enzyme Gpx4 can eliminate lipid peroxides in the mitochondria to inhibit the production of Cyt-c and reduce apoptosis (73). DQ can also affect the caspase signal pathway by inducing oxidative stress to produce ROS and enhance the activities of caspase-3 and caspase-9 in the liver tissue (74–78). It is found that there is a relationship between Bax/Bcl-2 and caspase. With the increase of Bax/Bcl-2 ratio, caspases are also gradually activated, which can promote apoptosis (79). It was also found that the activity of mitochondrial complex I was inhibited and ATP was consumed, which proved the destructive effect of DQ on the mitochondria. In addition, it is found that mitochondrial dysfunction and reduced apoptosis by inhibiting NF-KB and p53 signal pathways are the main pathways for DQ to induce inflammation and apoptosis. Secondly, some studies have found that the release of Smac/Diablo, endonuclease G, and other intermembrane space proteins after permeation of mitochondrial outer membrane can promote cell apoptosis by activating cystatin. A large amount of H_2_O_2_ is produced by the mitochondria, which further induces the production of ROS to aggravate the oxidative stress of cells that leads to further apoptosis (80).

Our research results are somewhat consistent with the above research results. In our study, we did not find that DQ had significant effects on pro-apoptotic factors BAX, BAK1, TNF-α, caspase series, and p53, but they showed an upward trend all together. The anti-apoptotic genes *Parkin* and *Bcl2* were significantly upregulated, indicating that anti-apoptotic factors in ducklings play an important role in the period of acute DQ poisoning. It is well-known that the production of a large number of ROS can destroy the function of the mitochondria. Some studies have suggested that it may convert the apoptosis pathway into necrosis, thus showing the increase of inhibitory apoptosis factors. Previous studies have shown that *Parkin* gene not only plays an anti-apoptotic role but also inhibits the pro-apoptotic pathway of p53 (81, 82). In our study of autophagy factor, we found that DQ significantly upregulated the protein expression of autophagy factor Beclin 1 in the liver. The key autophagy factors LC3A, LC3B, and p62 showed an upward trend at the gene level, but the protein expression of LC3B and p62 decreased; it may be due to the effect of pro-apoptotic components such as apoptotic proteases during translation, which initiated apoptosis and inhibited the occurrence of autophagy at the same time (62). Most evidence shows that autophagy is the protective mechanism of cell initiation. Autophagy can inhibit apoptosis when autophagy is upregulated; similarly, apoptosis can also reduce autophagy. Therefore, we believe that acute duck poisoning has a significant effect on the liver. On the one hand, it activates the apoptosis and anti-apoptosis system, which proves that there is apoptosis in hepatocytes. On the other hand, the autophagy protection system was activated, and it was found that the autophagy and apoptosis system inhibited each other.

## Data Availability Statement

The raw data supporting the conclusions of this article will be made available by the authors, without undue reservation.

## Ethics Statement

The animal study was reviewed and approved by Animal Ethics Committee of South China Agricultural University (License Number: 2017A087).

## Author Contributions

JC, YS, RL, and FL were responsible for the study conception and design. RH and DS revised the manuscript. JC, YS, PS, and DS were involved in the drafting of the manuscript. All authors contributed to the article and approved the submitted version.

## Conflict of Interest

The authors declare that the research was conducted in the absence of any commercial or financial relationships that could be construed as a potential conflict of interest.

## Publisher's Note

All claims expressed in this article are solely those of the authors and do not necessarily represent those of their affiliated organizations, or those of the publisher, the editors and the reviewers. Any product that may be evaluated in this article, or claim that may be made by its manufacturer, is not guaranteed or endorsed by the publisher.
